# Synergistic association of high-sensitivity C-reactive protein and body mass index with insulin resistance in non-diabetic adults

**DOI:** 10.1038/s41598-020-75390-1

**Published:** 2020-10-28

**Authors:** Gyu Ri Kim, Dong-Woo Choi, Chung Mo Nam, Sung-In Jang, Eun-Cheol Park

**Affiliations:** 1 Department of Preventive Medicine, College of Medicine, Yonsei University, Seoul, Korea; 2 Department of Public Health, Graduate School, Yonsei University, Seoul, Korea; 3 Department of Biostatistics, College of Medicine, Yonsei University, Seoul, Korea; 4 Institute of Health Services Research, Yonsei University, Seoul, Korea

**Keywords:** Endocrine system and metabolic diseases, Endocrinology, Risk factors, Epidemiology

## Abstract

Epidemiological evidence has indicated that inflammatory markers and obesity are strongly correlated with insulin resistance (IR). However, there is a paucity of studies assessing the complex interaction between elevated hs-CRP and body mass index (BMI), particularly among Asians. This study investigated the additive interaction between hs-CRP and BMI on IR, using cross-sectional data from the 7th Korea National Health and Nutrition Examination Survey (2016–2018). A total of 5706 men and 6707 women aged 20 years or older were evaluated, and a multiple logistic regression analysis was used to assess the association of serum hs-CRP and BMI with IR, as measured by the triglyceride-glucose index (TyG index). Sex-specific median values were used to dichotomise the continuous TyG index variable into insulin-sensitive and IR categories. Biological interaction was evaluated using the Relative excess risk due to interaction (RERI), attributable proportion due to interaction (AP), and synergy index (SI). The joint effects of high hs-CRP and overweight/obesity on IR were greater than would be expected from the effects of the individual exposures alone. Relative to those with low hs-CRP and BMI < 23, having both exposures was related to increased IR with an adjusted OR of 2.97 (95% CI 2.50–3.52) in men and 3.08 (95% CI 2.67–3.56) in women with significant additive interactions. These findings demonstrate that IR prevention strategies that reduce both systematic inflammation and BMI may exceed the expected benefits based on targeting these risk factors separately.

## Introduction

Insulin resistance (IR) is a condition characterised by the inability of the peripheral tissues to properly utilize endogenous insulin to maintain glucose homoeostasis. Several lines of evidence suggest that IR is an underlying cause of a range of health outcomes, including cardiovascular diseases^1,2^, cognitive dysfunction^3^, frailty^4^, and certain cancers^5,6^. IR has also been established as a major feature of the development and pathophysiology of type 2 diabetes (T2DM)^7,8^. It represents one of the leading causes of premature mortality in the adult population globally, with an estimated 1.6 million deaths caused by diabetes^9^. Further, the International Diabetes Federation (IDF) projected that by 2045, the number of people diagnosed with diabetes will increase by 51%, to 700 million^10^. Consequently, the disease burden associated with diabetes will continue to escalate over the next decades.

In light of the high morbidity and mortality rates associated with T2DM, prominent scientific efforts have been directed towards identifying possible risk factors for insulin resistance. Accumulating experimental and epidemiologic evidence has shown that inflammatory processes are linked to impaired glucose metabolism. In particular, the systemic inflammatory biomarker C-reactive protein (CRP), when measured in the blood with high sensitivity assay, has been reported to highly correlate with insulin resistance in diabetic^11^ and non-diabetic individuals^12,13^. IR is known to be associated with chronic inflammation, which is induced by various pro-inflammatory cytokines and oxidative stress biomarkers, notably interleukin-1 beta (IL-1β), interleukin-6 (IL-6), and adipocytokines^14,15^. Chronic exposure to elevated levels of pro-inflammatory biomarkers stimulates the activation of cytokine signalling pathways, which prevent the activation of insulin signalling receptors in β-cells of pancreatic islets. In addition, obesity is closely related to insulin resistance. Furthermore, dysfunctional lipid metabolism accompanies obesity and can negatively regulate insulin action. Few studies, however, have assessed the combined association of increased hs-CRP and body mass index (BMI) on insulin resistance, particularly in Asian populations. Therefore, we sought to investigate this relationship in a large representative sample of non-diabetic adults from the 2016–2018 Korea National Health and Nutrition Examination Survey.

## Results

### Participant characteristics

The characteristics of 5706 men and 6707 women, according to insulin resistance status, who were included in this study are shown in Table 1. Among all participants, 45.97% were men, and the prevalence of insulin resistance was 50.04% and 50.02% in men and women, respectively. In both men and women, the insulin-resistant group had a significantly higher proportion of overweight/obese individuals, smokers, and drinkers than the insulin-sensitive group. Furthermore, a lower prevalence of hypertension and dyslipidemia was noted in the insulin-sensitive group. Table 2 outlines the general characteristics of participants with serum hs-CRP levels above and below the median value. Overall, individuals with high hs-CRP were more likely to be older, rural residents, of lower household incomes, less educated, overweight/obese, current smokers, and drinkers than those with hs-CRP values below the median. Moreover, individuals with high hs-CRP levels had a higher prevalence of hypertension and dyslipidemia and a lower physical activity level than their counterparts with lower hs-CRP levels.

**Table 1:** General characteristics of the study population by insulin resistance status, as estimated by TyG index, Korea National Health and Nutrition Examination Survey 2016–2018.

	Insulin resistance (TyG index^a^)													
	Men (n = 5706)						*P* value*	Women (n = 6707)						*P* value*
	Total		No (< 8.706)		Yes (≥ 8.706)			Total		No (< 8.355)		Yes (≥ 8.355)		
	N	%	N	%	N	%		N	%	N	%	N	%	
**Serum hs-CRP level**							**< 0.0001**							**< 0.0001**
Low	2715	47.58	1564	57.61	1151	42.39		3343	49.84	2046	61.20	1297	38.80	
High	2991	52.42	1287	43.03	1704	56.97		3364	50.16	1306	38.82	2058	61.18	
**Age in years**							**< 0.0001**							**< 0.0001**
20–29	781	13.69	518	66.33	263	33.67		880	13.12	656	74.55	224	25.45	
30—39	1101	19.30	537	48.77	564	51.23		1315	19.61	835	63.50	480	36.50	
40—49	1153	20.21	465	40.33	688	59.67		1525	22.74	809	53.05	716	46.95	
50—59	1037	18.17	445	42.91	592	57.09		1367	20.38	562	41.11	805	58.89	
≥ 60	1634	28.64	886	54.22	748	45.78		1620	24.15	490	30.25	1130	69.75	
**Monthly household income**							**0.070**							**< 0.0001**
Low	788	13.81	421	53.43	367	46.57		989	14.75	349	35.29	640	64.71	
Mid-low	1325	23.22	661	49.89	664	50.11		1638	24.42	740	45.18	898	54.82	
Mid-high	1677	29.39	849	50.63	828	49.37		1963	29.27	1021	52.01	942	47.99	
High	1916	33.58	920	48.02	996	51.98		2117	31.56	1242	58.67	875	41.33	
**Educational level**							**0.001**							**< 0.0001**
High school diploma or below	3093	54.21	1594	51.54	1499	48.46		3960	59.04	1665	42.05	2295	57.95	
College graduate or above	2613	45.79	1257	48.11	1356	51.89		2747	40.96	1687	61.41	1060	38.59	
**Region**							0.974							**0.0002**
Urban	4732	82.93	2361	49.89	2371	50.11		5675	84.61	2892	50.96	2783	49.04	
Rural	974	17.07	490	50.31	484	49.69		1032	15.39	460	44.57	572	55.43	
**BMI (kg/m**^**2**^**)**							**< 0.0001**							**< 0.0001**
Underweight or normal (< 23.0)	1899	33.28	1260	66.35	639	33.65		3566	53.17	2265	63.52	1301	36.48	
Overweight or obese (≥ 23.0)	3807	66.72	1591	41.79	2216	58.21		3141	46.83	1087	34.61	2054	65.39	
**Smoking status**							**< 0.0001**							**< 0.0001**
Non-smoker	3637	63.74	1990	54.72	1647	45.28		6303	9398	3196	50.71	3107	49.29	
Current smoker	2069	36.26	861	41.61	1208	58.39		404	6.02	156	38.61	248	61.39	
**Drinking status**							**< 0.0001**							**<0.0001**
Non-drinker	677	11.86	394	58.20	283	41.80		1405	20.95	595	42.35	810	57.65	
Drinker	5029	88.14	2457	48.86	2572	51.14		5302	79.05	2757	52.00	2545	48.00	
**Physical activity**							**0.001**							**< 0.0001**
Active	2740	48.02	1430	52.19	1310	47.81		2939	43.82	1598	54.37	1341	45.63	
Inactive	2966	51.98	1421	47.91	1545	52.09		3768	56.18	1754	46.55	2014	53.45	
**Hypertension**							**< 0.0001**							**< 0.0001**
No	3901	68.37	2115	54.22	1786	45.78		5280	78.72	2951	55.89	2329	44.11	
Yes	1805	31.63	736	40.78	1069	59.22		1427	21.28	401	28.10	1026	71.90	
**Dyslipidemia**							**< 0.0001**							**< 0.0001**
No	3228	56.57	2189	67.81	1039	32.19		3479	51.87	2379	68.38	1100	31.62	
Yes	2478	43.43	662	26.72	1816	73.28		3228	48.13	973	30.14	2255	69.86	
**Cardiovascular diseases**							0.207							**< 0.0001**
No	5438	95.30	2707	49.78	2731	50.22		6552	97.69	3312	50.55	3240	49.45	
Yes	268	4.70	144	53.73	124	46.27		155	2.31	40	25.81	115	74.19	
**Total participants**	5706	100.0	2851	49.96	2855	50.04		6707	100.00	3352	49.98	3355	50.02	

Boldface *P*-values indicate statistical significance.

ªTyG index was calculated by the formula ln[fasting triglycerides (mg/dL) × fasting glucose (mg/dL)/2].

*P values were obtained by Chi-square test or Fisher’s exact test.

**Table 2:** General characteristics of the study population according to sex-specific median hs-CRP levels.

	Serum hs-CRP level									
	Men (n = 5706)				*P* value*	Women (n = 6707)				*P* value*
	Low (< 0.60 mg/L)		High (≥ 0.60 mg/L)			Low (< 0.50 mg/L)		High (≥ 0.50 mg/L)		
	N	%	N	%		N	%	N	%	
**Age in years**					**< 0.0001**					**< 0.0001**
20–29	454	16.72	327	10.93		534	15.97	346	10.29	
30–39	527	19.41	574	19.19		704	21.06	611	18.16	
40–49	575	21.18	578	19.32		840	25.13	685	20.36	
50–59	451	16.61	586	19.60		632	18.91	735	21.85	
≥ 60	708	26.08	926	30.96		633	18.94	987	29.34	
**Monthly household income**					** < 0.0001**					** < 0.0001**
Low	313	11.53	475	15.88		392	11.73	597	17.75	
Mid-low	604	22.25	721	24.11		765	22.88	873	25.95	
Mid-high	829	30.53	848	28.35		1007	30.12	956	28.42	
High	969	35.69	947	31.66		1179	35.27	938	27.88	
**Educational level**					** < 0.0001**					** < 0.0001**
High school diploma or below	1385	51.01	1708	57.10		1850	55.34	2110	62.72	
College graduate or above	1330	48.99	1283	42.90		1493	44.66	1254	37.28	
**Region**					**0.001**					**0.0004**
Urban	2297	84.60	2435	81.41		2881	86.18	2794	83.06	
Rural	418	15.40	556	18.59		462	13.82	570	16.94	
**BMI (kg/m**^**2**^**)**					** < 0.0001**					** < 0.0001**
Underweight or normal (< 23.0)	1121	41.29	778	26.01		2264	67.72	1302	38.70	
Overweight or obese (≥ 23.0)	1594	58.71	2213	73.99		1079	32.28	2062	61.30	
**Smoking status**					**0.003**					0.243
Non-smoker	1785	65.75	1852	61.92		3153	94.32	3150	93.64	
Current smoker	930	34.25	1139	38.08		190	5.68	214	6.36	
**Drinking status**					0.083					** < 0.0001**
Non-drinker	301	11.09	376	12.57		597	17.86	808	24.02	
Drinker	2414	88.91	2615	87.43		2746	82.14	2554	75.98	
**Physical activity**					**0.022**					**0.001**
Active	1347	49.61	1393	46.57		1536	45.95	1403	41.71	
Inactive	1368	50.39	1598	53.43		1807	54.05	1961	58.29	
**Hypertension**					** < 0.0001**					** < 0.0001**
No	1976	72.78	1925	64.36		2827	84.56	2453	72.92	
Yes	739	27.22	1066	35.64		516	15.44	911	27.08	
**Dyslipidemia**					** < 0.0001**					** < 0.0001**
No	1707	62.87	1521	50.85		2097	62.73	1382	41.08	
Yes	1008	37.13	1470	49.15		1246	37.27	1982	58.92	
**Cardiovascular diseases**					0.346					**0.008**
No	2595	95.58	2843	95.05		3286	98.29	3266	97.09	
Yes	120	4.42	148	4.95		57	1.71	98	2.91	

Boldface *P*-values indicate statistical significance.

*P values were obtained by Chi-square test or Fisher's exact test.

### Association of insulin resistance with hs-CRP levels and BMI

The age- and multivariable-adjusted odds ratios (ORs) and 95% confidence intervals (CI) for the associations of hs-CRP level or BMI with insulin resistance by sex are shown in Table 3. In the multivariable-adjusted analyses, serum hs-CRP and BMI were independently associated with insulin resistance, after controlling for age, region, monthly household income, educational level, smoking status, drinking status, physical activity, hypertension, dyslipidemia, and self-reported diagnosis of cardiovascular diseases. In the comparison of high versus low hs-CRP levels, elevated serum hs-CRP levels, above the median, were associated with a 1.57-fold (OR 1.57, 95%CI 1.39–1.76) and 1.84-fold (OR 1.84, 95% CI 1.65–2.05) increased OR of IR in men and women, respectively. Furthermore, in both men and women, higher BMI was associated with higher odds of being insulin resistant. Men with a BMI ≥ 23.0 had a 2.26-fold greater OR of becoming insulin resistant, compared to the reference group (OR: 2.26, 95% CI 1.98.04–2.57). The corresponding adjusted OR for IR in women was 2.14 (95% CI 1.91–2.39).

**Table 3:** Age- and multivariable-adjusted ORs and 95% CIs for insulin resistance according to serum high-sensitivity CRP level and BMI in separate models, KNHANES 2016–2018.

		Age adjusted^a^	Multivariable adjusted^b^
		OR (95% CI)	OR (95% CI)
**Men**	**Serum hs-CRP**		
	Low	1.00	1.00
	High	1.78 (1.60–1.98)	1.57 (1.39–1.76)
	**BMI**		
	Underweight or normal (< 23.0)	1.00	1.00
	Overweight or obese (≥ 23.0)	2.65 (2.36–2.97)	2.26 (1.98–2.57)
**Women**	**Serum hs-CRP**		
	Low	1.00	1.00
	High	2.26 (2.04–2.51)	1.84 (1.65–2.05)
	**BMI**		
	Underweight or normal (< 23.0)	1.00	1.00
	Overweight or obese (≥ 23.0)	2.74 (2.47–3.04)	2.14 (1.91–2.39)

^a^Adjusted for age.

^b^Adjusted for age, region, household income, educational level, smoking status, drinking status, physical activity, hypertension, dyslipidemia, cardiovascular disease.

*OR* odds ratio, *CI* confidence interval, *BMI* body mass index.

### Synergistic interaction of elevated hs-CRP levels and BMI on insulin resistance

**Figure 1:**
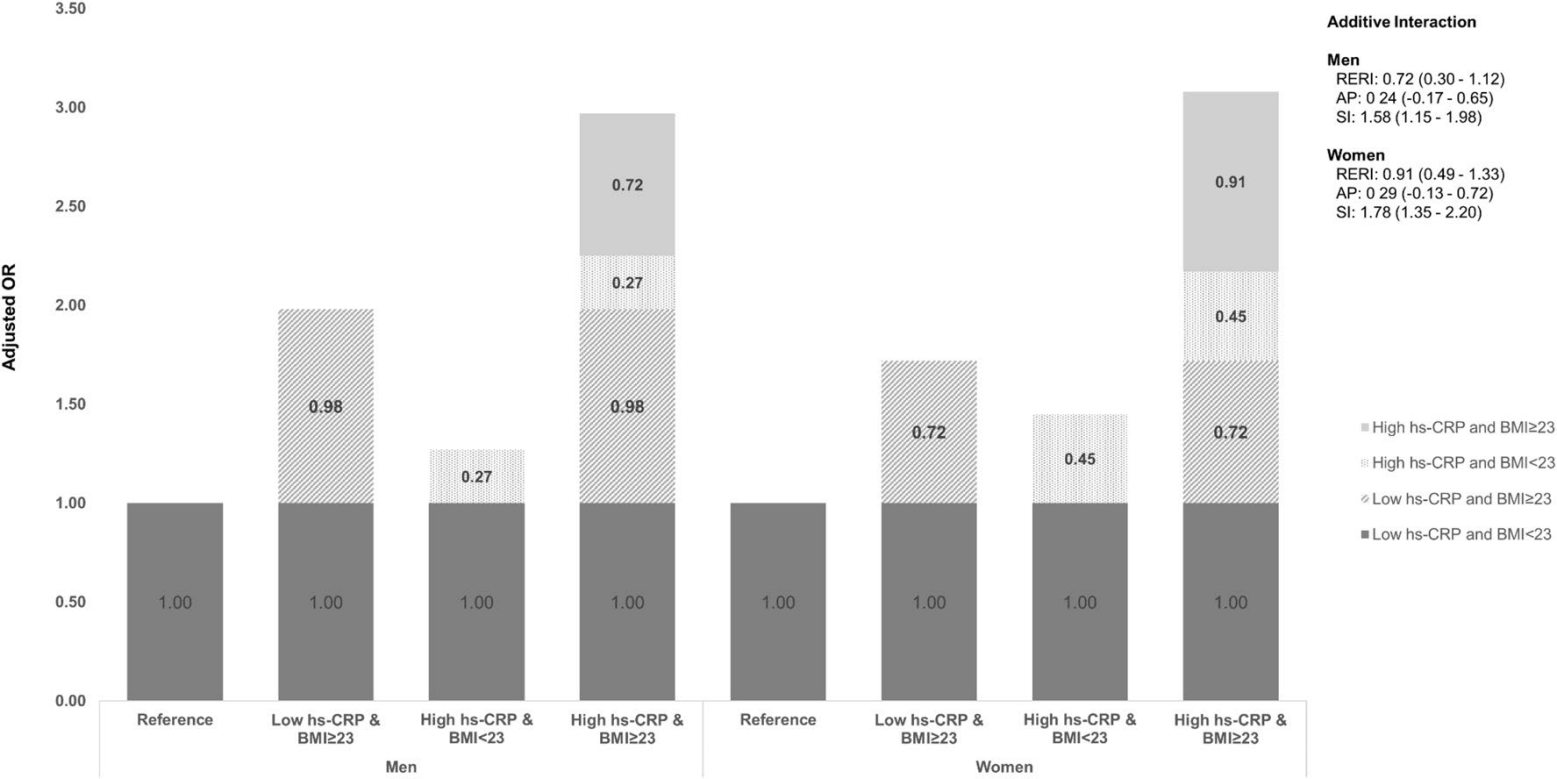
Additive interaction of high hs-CRP and BMI on insulin resistance.

As shown in Fig. 1, we found a statistically significant synergistic interaction between hs-CRP and body mass index on insulin resistance. In the multivariable analysis and using men with low hs-CRP and BMI < 23 as the reference category, BMI ≥ 23 alone or high hs-CRP alone were associated with increased odds of insulin resistance (OR 1.98; 95% CI 1.66–2.37 and OR 1.27; 95% CI 1.03–1.58, respectively). Furthermore, having both risk factors greatly enhanced the OR of insulin resistance to 2.97 (95% CI 2.50–3.52). The estimated RERI was 0.72 (95% CI 0.30–1.12), indicating a synergistic interaction of hs-CRP and overweight/obesity on insulin resistance. In other words, the OR for the concurrence of obesity and elevated hs-CRP levels on insulin resistance was beyond the sum of the odds ratios associated with their individual effect. In addition, the attributable proportion (AP) revealed that 24% of the total odds of being insulin resistance was related to the interaction between elevated hs-CRP and BMI. Additionally, the synergy index (SI 1.58, 95% CI 1.15–1.98) also confirmed a synergistic interaction. Among women, the OR for the combination of high hs-CRP and BMI ≥ 23 (OR 3.08; 95% CI 2.67–3.56) was greater than the ORs for high hs-CRP and BMI < 23 (OR 1.45; 95% CI 1.24–1.69) and low hs-CRP and BMI ≥ 23 (OR 1.72; 95% CI 1.46–2.03). The RERI for insulin resistance was statistically significant in women (RERI 0.91; 95% CI 0.49–1.33). The AP was 0.29 (95% CI 0.13–0.72), and the SI was 1.78 (95% CI 1.35–2.20).

### Sensitivity analysis

In order to evaluate the robustness of these associations, a sensitivity analysis was conducted, in which we excluded participants with hypertension, dyslipidemia, and self-reported diagnosis of cardiovascular diseases. As shown in Supplementary Table S1, the magnitude and direction of the association remained essentially unchanged in the sensitivity analysis. However, due to the smaller sample size the statistical power was limited for the interaction analyses, and additive interaction measures did not reach statistical significance^16^.

## Discussion

In this population-based cross-sectional study of Korean non-diabetic participants aged 20 years or older, we found that individuals with elevated hs-CRP levels were at significantly higher odds of having insulin resistance, and that these associations persisted, even after adjusting for other relevant socio-demographic and health-related variables. Thus, our study confirms that systemic inflammation due to high hs-CRP levels has an independent effect on insulin resistance, as estimated by the TyG index.

The euglycemic hyperinsulinemic clamp method is considered the gold standard for estimating IR; however, because it is expensive and invasive, other surrogate IR indices have been developed based on anthropometric or biochemical parameters that are routinely collected in clinical practise. One such measure is the TyG index, which has been previously shown to be a simple, efficient, and clinically useful surrogate marker of IR. Two recent studies have shown that the TyG index is closely correlated with the homoeostasis model assessment of insulin resistance index (HOMA-IR)^17,18^. Moreover, other studies have reported that the TyG index value is superior for predicting IR to that of HOMA-IR^18–20^.

Elevated serum hs-CRP levels provide a sensitive marker of subclinical inflammation. Our finding of a positive association between serum hs-CRP levels and IR has been supported by several studies that have reported an association between CRP concentrations and surrogate measures of insulin resistance such as HOMA-IR and quantitative insulin sensitivity check index (QUICKI)^21–23^. The longitudinal cohort from the Bogalusa Heart Study of black and white adults showed that elevated hs-CRP at baseline was associated with future insulin resistance, as measured by HOMA-IR, after accounting for race, sex, age, BMI, smoking, alcohol drinking, and follow-up years^24^. This is also consistent with previous studies that reported associations between hs-CRP and insulin resistance among specific populations. In an occupational cohort of 10,308 British civil servants in the Whitehall II study, baseline subclinical inflammation biomarker levels were related to subsequent five-year changes in IR among non-diabetic individuals^25^. In contrast, a cross-sectional study of men aged 40–80 years did not find a significant association between CRP level and insulin resistance, after controlling for body composition parameters.

Additionally, we observed that the combined effects of systemic inflammation and BMI were greater than the sum of their individual impacts. Relative to individuals with low hs-CRP and BMI < 23, those with concomitant systemic inflammation and overweight/obese status had up to three-times greater odds of being insulin resistant. In our study, there was a relatively modest synergistic interaction between elevated serum hs-CRP levels and overweight/obese status on the OR of insulin resistance, and approximately 24% and 29% of effects were attributable to additive interactions in men and women, respectively. These results are in accordance with the findings of an earlier study that identified a significant interaction between body size and serum hs-CRP on insulin resistance in a cohort of Japanese adults aged 35–69 years^23^.

Mechanistically, systemic inflammation and obesity may affect insulin resistance via several pathways. Notably, fat accumulation in the liver or adipose tissues has been found to induce the production of pro-inflammatory cytokines, such as tumour necrosis factor alpha (TNF-α) and IL-6^26,27^. These adipocyte-produced cytokines stimulate hepatic CRP production and activation of the innate immune system^28,29^. An in vivo in mice found that chronic exposure to IL-6 impairs insulin receptor signalling in primary hepatocytes^30^. Therefore, increased TNF-α and/or IL-6 secretion may be responsible for the observed relationship between elevated serum hs-CRP and IR. Furthermore, inflammation may promote the development of IR by triggering the development of hypertension by influencing platelet adhesion, aggregation, and oxidant production^31^. Moreover, prior studies suggest that subclinical inflammation leads to diminished nitric oxide synthesis in endothelial cells and endothelial dysfunction, which promotes IR^32–35^. Additional studies are required to clarify the precise mechanisms and establish the causal effects of systemic inflammation and obesity on insulin sensitivity.

The present study has several strengths. First, this study features a nationally representative sample that surveyed a large sample of Korean adults using standardised questionnaires and laboratory procedures. Hence, the findings are likely to be generalizable to the overall Korean population. Furthermore, we attempted to minimise potential bias due to reverse causation by excluding participants with a diabetes and those who use anti-diabetic medication. However, our results should be considered in light of certain limitations. First, important questions that arises from these observations is whether adiposity-induced inflammation precedes IR or vice versa, and whether this relationship is bidirectional, particularly in the pathophysiology of T2DM. A cross-sectional study design with a single assessment of hs-CRP limits inferences regarding the temporal relationship between hs-CRP and IR. Therefore, prospective cohort studies may provide a better context for answering these questions. Second, the self-reporting of health-related factors and history of cardiovascular diseases could have led to recall bias. Lastly, although a wide spectrum of confounders was included in the adjustments, the presence of certain residual or undetected confounding factors cannot be completely ruled out.

In summary, our study provides further evidence that concomitant systemic inflammation and overweight/obesity may synergistically contribute to IR, such that prevention strategies for IR that aim to reduce both systematic inflammation and BMI may exceed the benefits that are expected from targeting these risk factors separately. These findings need to be confirmed in future longitudinal prospective studies to establish causality between systemic inflammation, obesity, and IR.

## Material and methods

### Study population

The current study is based on data from the 2016–2018 Korean National Health and Nutrition Examination Survey (KNHANES)^36^. In brief, the KNHANES is a population-based cross-sectional study designed to assess the health and nutritional status of people residing in South Korea. The survey included a stratified multistage probability sample that is representative of the non-institutionalized civilian population, aged one year or above. The three major components of the KNHANES are health interviews, health examinations, and nutrition surveys. The KNHANES interview includes detailed questions on demographic, socioeconomic, dietary, and health-related characteristics. The health examination component comprises medical, dental, and physiological measurements, as well as laboratory tests administered by trained medical personnel. The KNHANES survey protocols were approved by the Institutional Review Board of the Korea Centers for Disease Control and Prevention (IRB No. 2018-01-03-P-A), and the study complied with the Declaration of Helsinki for medical research involving human subjects. Informed consent was obtained from all participants.

Of the 24,269 participants (men: 11,071; women: 13,198) who participated in the 2016–2018 survey, we restricted our analysis to include adults aged 20 years or older. Exclusion criteria included those with a previous diagnosis of diabetes, individuals who were using anti-diabetic medication, or pregnant women (N = 16,638, men: 7198; women: 9440). After excluding those with missing data on IR status or other covariates, 12,413 participants (men: 5706; women: 6707) were analysed.

### Definition of insulin resistance

The main study outcome was IR, as measured by the triglyceride-glucose index (TyG index)^37^. The TyG index was calculated according to the following formula: log [fasting triglyceride (mg/dL) × fasting glucose (mg/dL)/2]. The TyG index has been proposed as a simple and clinically useful surrogate for IR^18,38,39^. As no cut-off points have been suggested in the literature, sex-specific median values (men: 8.076, women: 8.355) were used to dichotomise the continuous TyG index variable into insulin-sensitive and insulin-resistant groups.

### Laboratory measures

Blood samples were drawn from the antecubital vein in the morning of examinees who had fasted for at least eight hours. Serum hs-CRP levels were measured using an immunoturbidimetric assay on a Roche Cobas analyser. The subjects were dichotomised into “high” and “low” categories, based on sex-specific median hs-CRP values. The median hs-CRP cut-off values were 0.60 mg/L in men and 0.50 mg/L in women.

All other biochemical parameters, including triglycerides (TG), total cholesterol (TC), and fasting blood glucose (FBG) were measured immediately and detected enzymatically using an automatic chemistry analyser (Hitachi 7600, Hitachi Ltd., Tokyo, Japan). Low-density lipoprotein cholesterol (LDL-C) and high-density lipoprotein cholesterol (HDL-C) levels were measured using homogeneous enzymatic colourimetric methods.

### Other covariates

All potential confounding variables were selected based on prior knowledge of the existing literature^40–42^. Standardised questionnaires were administered by well-trained interviewers to collect information on age, sex, socioeconomic characteristics, and health-related risk factors. Participants, based on age, were categorised into the following groups: 20–29, 30–39, 40–49, 50–59, and ≥ 60 years. Participants were also categorised by educational attainment (High school diploma or below and college graduate or above) and monthly household income quartiles. Residential areas were divided into urban and rural areas.

Information on smoking status, drinking status, and physical activity was also obtained. Smoking status was categorised as either non-smoker (including never- and former-smokers) or current smoker. Participants were asked about alcohol consumption as follows: In the past year, how often did you drink any type of alcoholic beverage? Individuals were considered current drinkers if they consumed at least one drink in the past year. Physical activity was defined as engagement in 150 min of moderate-intensity aerobic activity or 75 min of vigorous-intensity aerobic activity every week.

In KNHANES, anthropometric data were collected by well-trained personnel according to a standardised protocol. Body mass index (BMI [kg/m^2^]) was calculated using measurement of height and weight. A BMI < 23.0 was considered normal weight and a BMI greater than or equal to 23.0 was considered overweight/obese, according to the Asia–Pacific obesity classification^43^. Following the 2015 Korean Guidelines, dyslipidemia (yes/no) was defined as having one or more of the following lipid abnormalities: hypercholesterolemia (total cholesterol ≥ 240 mg/dL or use of lipid-lowering drugs), hypertriglyceridemia (TG ≥ 200 mg/dL), hyper-low density lipoprotein (LDL) cholesterolemia (≥ 160 mg/dL), and hypo-high density lipoprotein (HDL)-cholesterolemia (< 40 mg/dL in men and < 50 mg/dL in women)^44^. Hypertension (yes/no) was defined as a systolic blood pressure of 140 mmHg or higher, diastolic blood pressure of 90 mmHg or higher, or on antihypertensive treatment. Based on self-report, adults who answered ‘yes’ to the following questions were also ascertained as having cardiovascular diseases: ‘Has a doctor or other health professional told you have had a stroke, angina, or myocardial infarction?”.

### Statistical analyses

Statistical analyses were conducted using SAS version 9.4 (SAS Institute Inc., Cary, NC, USA). For descriptive purposes, variables were summarised in frequencies, and sample proportions of categorical variables were obtained. Chi-square or Fisher's exact tests were used to determine whether the distribution of categorical covariates of insulin-sensitive participants differed from the distributions of insulin-resistant individuals. To examine associations of hs-CRP and body mass index with IR, odds ratios (ORs) and 95% confidence intervals were estimated using logistic regression. Age, region, monthly household income, educational level, smoking status, drinking status, physical activity, hypertension, dyslipidemia, and self-reported diagnosis of cardiovascular diseases were adjusted, when appropriate.

Additionally, we investigated the presence of biological interactions on an additive scale, that is, whether the combination of high hs-CRP and BMI poses greater risk than the sum of their independent effects. This was evaluated using three indices of additive interaction: RERI (the relative excess risk due to interaction), AP (the proportion attributable to interaction), and the synergy index (SI)^45,46^. The RERI is defined as the additional risk due to interaction, which is calculated as the difference between the expected risk based on the addition of the ORs of the separate effects of the two risk factors under exposure and the observed risk in the doubly exposed group: RERI = OR_11_-OR_10_-OR_01_ + 1, where OR_11_ refers to the odds ratio of insulin resistance for high hs-CRP combined with BMI ≥ 23; OR_10_ is the OR of insulin resistance for elevated serum hs-CRP with BMI < 23; and OR_01_ represents exposure to BMI ≥ 23 only. The AP is interpreted as the proportion of disease that is due to interaction among individuals with both exposures: AP = RERI/OR_11_. The SI refers to the excess risk from both exposures when there is interaction, relative to the risk from exposure without interaction: SI = (OR_11_–1)/[(OR_01_–1) + (OR_10_–1)]). The resulting confidence intervals of RERI > 0 or AP > 0 or SI > 1 were indicative of positive departure from additivity, or synergistic interaction of the effects from two exposures on insulin resistance. In these analyses, adjustments were made for the same sets of potential confounders as described for the logistic regression models. All statistical test with a 2-tailed P < 0 0.05 were considered statistically significant.

## Supplementary information

Supplementary Information
