# Supplementary material for: Synergistic association of high-sensitivity C-reactive protein and body mass index with insulin resistance in non-diabetic adults
Source: Sci Rep. 2020 Oct 28;10:18417. doi: 10.1038/s41598-020-75390-1 (PMC7595183; doi:10.1038/s41598-020-75390-1)
Supplement: Supplementary file 1 — Supplementary Information [file 41598_2020_75390_MOESM1_ESM.docx]

**Synergistic Association of High-sensitivity C-reactive protein and Body Mass Index with Insulin Resistance in Non-Diabetic Adults**

Gyu Ri Kim^1,4^, Dong-Woo Choi^2,4^, Chung Mo Nam^1,3^, Sung-In Jang^1,4^, Eun-Cheol Park^1,4*^

^1^Department of Preventive Medicine, College of Medicine, Yonsei University, Seoul, Korea.

^2^Department of Public Health, Graduate School, Yonsei University, Seoul, Korea.

^3^Department of Biostatistics, College of Medicine, Yonsei University, Seoul, Korea.

^4^Institute of Health Services Research, Yonsei University, Seoul, Korea

**Correspondence:** ecpark@yuhs.ac

Department of Preventive Medicine & Institute of Health Services Research, Yonsei University College of Medicine, Seoul, Republic of Korea

50-1 Yonsei-ro, Seodaemun-gu, Seoul, 03722, Korea

| **Supplementary Table S1. Results of the sensitivity analysis for combined effects of hs-CRP and BMI on IR after excluding participants with hypertension, dyslipidemia, and self-reported diagnosis of cardiovascular disease (N=5,363)** | | |  |  |  |
| --- | --- | --- | --- | --- | --- |
| **Categories** | **Men (N=2,348)** | **Women (N=3,015)** |  |  |  |
|  | **Adjusted OR (95% CI)** | **Adjusted OR (95% CI)** |  |  |  |
| Low-CRP & BMI<23 | 1.00 | 1.00 |  |  |  |
| Low-CRP & BMI≥23 | 2.17 (1.70-2.79) | 1.64 (1.25-2.15) |  |  |  |
| High-CRP &BMI<23 | 1.23 (0.95-1.60) | 1.33 (1.11-1.59) |  |  |  |
| High-CRP &BMI≥23 | 2.78 (2.20-3.49) | 2.27 (1.84-2.79) |  |  |  |
| RERI | 0.38 (-0.24 - 0.99) | 0.31 (-0.27 - 0.89) |  |  |  |
| AP | 0.14 (-0.48 - 0.75) | 0.13 (-0.45 - 0.72) |  |  |  |
| SI | 1.27 (0.65 - 1.89) | 1.32 (0.73 - 1.90) |  |  |  |
| RERI - Relative excess risk due to interaction; AP - attributable proportion due to interaction; SI - synergy index | | | | | |
| Models were adjusted for age, monthly household income, region, educational level, smoking status, drinking status, and physical activity. | | | | | |
